# Traditional bullying and cyberbullying in the digital age and its associated mental health problems in children and adolescents: a meta-analysis

**DOI:** 10.1007/s00787-022-02128-x

**Published:** 2022-12-31

**Authors:** Chao Li, Ping Wang, Marina Martin-Moratinos, Marcos Bella-Fernández, Hilario Blasco-Fontecilla

**Affiliations:** 1 Faculty of Medicine, Autonomous University of Madrid, Madrid, Spain; 2 Department of Psychiatry, Puerta de Hierro University Hospital, Health Research Institute Puerta de Hierro-Segovia de Arana (IDIPHISA), Majadahonda, Madrid, Spain; 3 Department of Psychology, Comillas Pontifical University, Madrid, Spain; 4 Center of Biomedical Network Research on Mental Health (CIBERSAM), Madrid, Spain; 5 Korian, ITA Mental Health, Madrid, Spain

**Keywords:** Traditional bullying, Cyberbullying, Suicidal ideations, Suicide attempts, Self-harm, Depression

## Abstract

Bullying is a risk factor for the physical and mental health of adolescents. The advent of new technologies has resulted in a brand-new type of bullying, cyberbullying (CB). The co-occurring effects of cyberbullying and traditional bullying(TB) forms of bullying on adolescent mental health are unclear. We performed a meta-analysis to explore the unique and combined effects of CB and TB on adverse psychological outcomes in victims by conducting a joint study of both types of bullying. By doing so, we provide the basis for a comprehensive community bullying prevention program. The database PubMed, PsyclNFO, and Web of Science were searched for studies from 2010 to 2021. The Preferred Reporting Items for Systematic Reviews and Meta-analyses (PRISMA) reporting guideline was followed for data abstraction, and the NIH tool was used to evaluate study-level risk of bias. 42 studies with 266,888 participants were identified. Random-Effect models were used for our study. The moderator analysis was used to explore the moderator of prevalence. Studies with three groups of victims (TB only, CB only, and Both) and two groups of victims (TB and CB) were compared in subgroup analysis. The mean victimization rate was 24.32% (95% CI 20.32–28.83%) for TB and 11.10% (95% CI 9.12–13.44%) for CB. Roughly one-third of TB victims were also victimized by CB. Conversely, only about one-third of CB victims were free from TB. The estimated ORs for depression, suicidal ideation, suicide attempts, and self-harm in the three-group (TB only, CB only and Both) analysis were: depression [TB only: 3.33 (2.22–5.00); CB only: 3.38 (2.57–4.46); Both: 5.30 (2.43–11.56)]; suicidal ideations [TB only: 3.08 (2.12–4.46); CB only: 3.52 (2.38–5.20); Both: 6.64 (4.14–10.64)]; self-harm [TB only: 2.70 (1.86–3.91); CB only: 3.57 (3.20–3.98); Both: 5.57 (2.11–16.00)]; and suicide attempts: [TB only: 2.61 (1.50–4.55); CB only: 3.52 (2.50–4.98); Both: 7.82 (3.83–15.93)]. TB and CB victimization among youth are a matter of public health concern. Victimization appears to be a marker of greater psychopathological severity, particularly suicide-related issues.

**Supplementary information:**

The online version of this article (10.1007/s00787-022-02128-x) contains supplementary material, which is available to authorized users.

## Introduction

Bullying is a significant risk factor for the physical and mental health of adolescents [1, 2]. In general, bullying is any unwanted aggressive behavior by other youths who are not siblings or current dating partners, that involves an observed or perceived power imbalance and is repeated recurrently or is highly likely to be repeated [3]. Traditional bullying (TB) has been face-to-face and included physical, verbal, or relational forms. With the development of internet technology, a new form of bullying, cyberbullying (CB), has emerged[4]. Previous meta-analyses have examined the issues related to TB and CB separately, suggesting that TB and CB overlapped[5]. However, few studies have investigated the extent of CB victimization and its prevalence relative to TB and whether the two types of bullying have different effects on the mental health of victims. In addition, there is no further updated research on whether the prevalence of CB and TB among victims has changed with the development of technology in the last decade.

With the constant evolution of communication technology, more children and adolescents have been exposed to smartphones in recent years. A study in 2018 [6] found that ninety-five percent of teens ages 13 to 17 said they had constant access to smartphones, and forty-five percent of these teens said they went online “almost constantly,” up from twenty-four percent in 2014. The Internet usage of adolescents has shifted from computer-based to mobile, providing convenience while increasing the amount of time spent online[7, 8]. According to the study by Hamm et al. [9], an increase in Internet use is associated with an increased frequency of CB.

However, the prevalence of CB varies widely across studies due to differences in definitions, measurements, and samples. According to a review, the prevalence of CB ranged from 4.8% to 73.5%, while another meta-analysis showed that cyber victimization rates ranged from 2.2 to 56.2% [5, 9]. There is also controversy regarding changes in the prevalence of CB [10]. The study of Modecki et al. [5] suggested that the prevalence of CB was likely overestimated due to concerns about the harmful consequences, but others, such as Hamm and Smith, argued that the prevalence of CB is increasing as the technology changes[4, 9]. On the other hand, CB and TB are considered highly correlated [9]. A recent large multinational study from 2002 to 2014 has concluded about samples from 37 countries that 45.8% of CB victims have also been bullied in real life [11]. The creation and quick adoption of new portable devices such as smartphones and tablets have significantly altered the communication and information environment during the past decade. Over 83% of UK teens aged 12 to 15 own their smartphones [12]. Based on the situation, a meta-analysis focusing on prevalence over the past decade might be a good reference.

In addition to prevalence, the correlation between TB and CB has also been a controversial point in recent years. Some studies [13–15] suggest that CB is similar to TB, both occur intentionally or repeatedly in situations of power imbalance. Another opinion considered CB as a distinct form of bullying, which is public, round-the-clock(7/24) and anonymous [10]. CB can be widely disseminated through messages, pictures, and videos, these make the perpetrators feel less guilty and act out-of-control while potentially increasing the number of bullies [10]. Although both TB and CB experiences may lead to many adverse psychological and social outcomes such as depression, anxiety, suicidal ideation, self-harm, low self-esteem, substance abuse, academic function and other health problems, the impact of these two types of bullying can be different [16–20]. Thus, victims of CB exhibit higher levels of anxiety and depression [21], as well as a higher risk of self-harm and suicidal behavior compared to victims of TB [22]. Although many studies have proposed that TB and CB are highly correlated [5, 9, 15, 20], research addressing the potential additive effect of both forms of bullying is insufficient.

Based on the status quo, it is necessary to recapitulate and explore: (1) the prevalence trends of TB and CB over the past decade, and (2) the co-occurring effects of both forms of bullying. To understand them better, the current meta-analysis will explore the unique and combined effects of CB and TB on several adverse psychological outcomes in victims by conducting a joint study of both types of bullying.

## Methods

Our study was guided by the Preferred Reporting Items for Systematic Reviews and Meta-Analyses (PRISMA) statement [23]. This meta-analysis aimed to examine the changes in prevalences of peer victimization of TB and CB during the mobile network era, and their association with mental health problems. The main mental health problems of interest were suicidal ideation, suicidal attempts, self-harm, and depression; others were analyzed if we got sufficient data from the results. Considering the heterogeneity of the measurement instruments and designs used in the studies, we also collected various feasible factors for a moderator analysis. To complete the included studies, the snowballing method was used during the article screening phase. This study was registered with the International Prospective Registry of Systematic Reviews (No. CRD42021250797).

### Search strategy

We searched PubMed, PsycINFO, and Web of Science databases for articles published from January 1, 2010, to April 12, 2021. Entering a combination of the following keywords: child, teenager, adolescent, bullying, cyberbullying, suicide, depression, self-harm, self-injurious behavior, mental health. The search was not restricted by language, country or type of research.

### Inclusion and exclusion criteria

- The study population was limited to children and youth ages 8 to 20; this included the age range of most studies on school bullying.
- All types of TB were included: verbal (e.g., name-calling and threats), physical (e.g., hitting) or psychological (e.g., rumors and shunning/exclusion). CB included someone making fun of another person online or picking on another person through social media, chat rooms or emails.
- Bullying behaviors primarily involved peers, excluding studies of bullying by siblings, parents, and teachers as perpetrators.
- Studies with clinical and primary care or incarcerated institutional samples, studies with ethnic minorities (e.g LGBTQ, disability) were excluded to ensure that the sample represented the situation of populations in a usual setting.
- To ensure the concordance of samples and comparability within studies of TB and CB. Included studies had to have self-report measures of peer victimization with both TB and CB; studies reporting only one type of bullying were excluded.
- These studies had to report the correlation of TB or CB victimization experiences with one of the following outcomes, such as self-harm, suicidal behavior (suicide attempts, suicidal ideation, or suicide plans), and mental health problems (depression, anxiety, etc.).
- The studies had to provide sufficient statistical information to calculate the necessary effect size (at least the prevalences of CB and TB victimization and one mental health problem) from the manuscript or after querying the authors. The effect sizes translated from the other measurements are acceptable.
- Studies had to be published either in English or Spanish. Book chapters, editorials, conference abstracts, letters to the editor, dissertations and posters were also eligible.

### Data selection and extraction

**Fig. 1:**
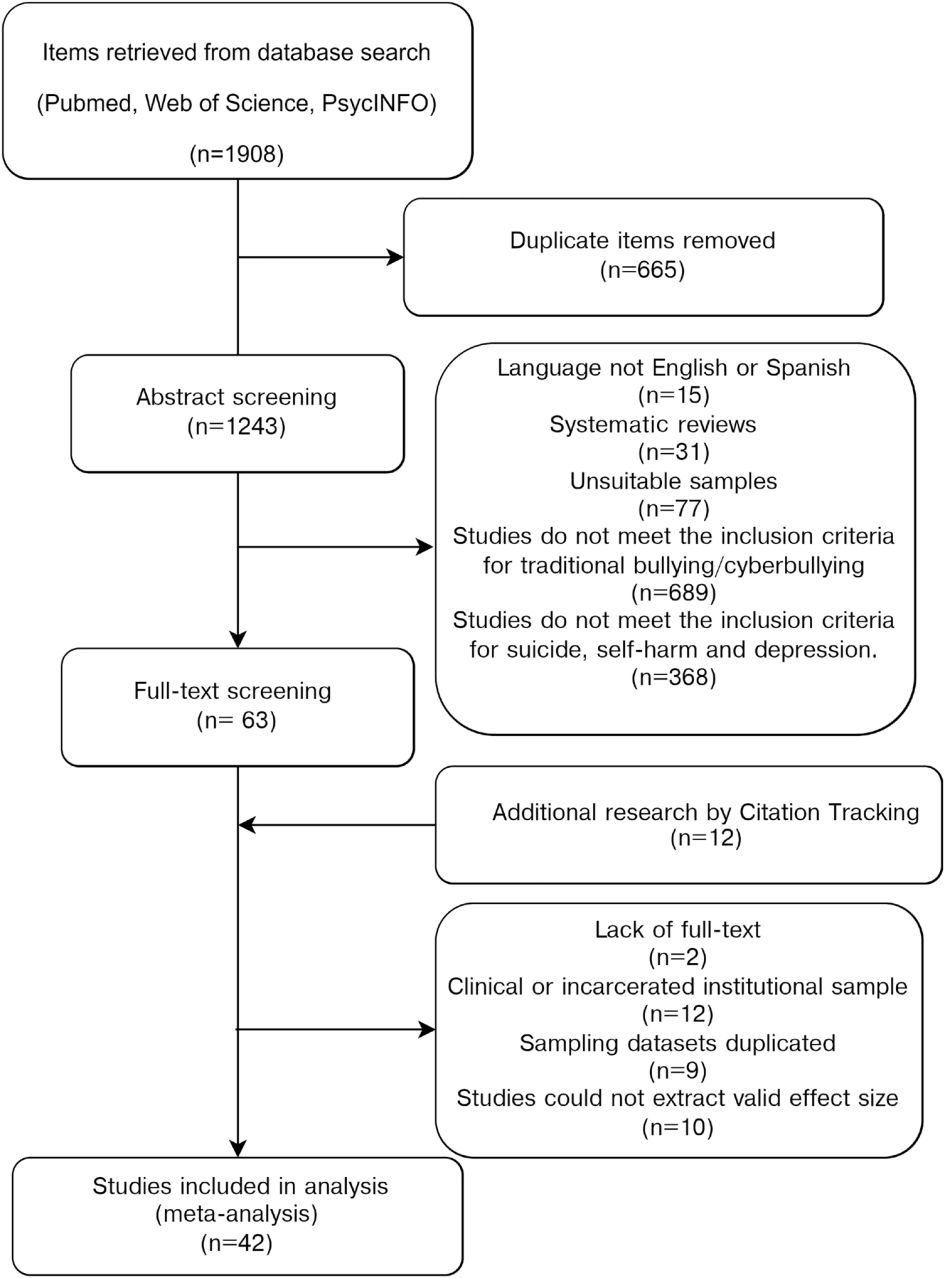
Flow diagram of all stages of the literature search

Three independent investigators (CL, PW and MM) screened all the research results by title and abstract, differences of opinion were resolved through discussion. Studies that matched the inclusion criteria were retrieved for full-text assessment. After discussion, a provisional coding book was created. Two authors (WP, CL) extracted data independently from included manuscripts. A third author (MM) resolved any disagreements in the extraction process. The extracted data included the author(s), year of publication, sampling countries, study objectives, study design, sample size, sample age distribution (included mean, standard deviation, and range of age), school grade level, measures of TB and CB (e.g., the questionnaires and number of items used in that study), measures of suicide, self-harm, and mental health problems(e.g., depression, anxiety) and other results. The flow diagram of our search results is provided in Fig. 1.

### Quality assessment

The quality of all articles was assessed using the “Quality Assessment Tool for Observational Cohort and Cross-Sectional Studies” provided by the National Institutes of Health (NIH) [24]. This tool determines an overall quality rating of good, fair and poor based on fourteen criteria. Given that most of the included articles were cross-sectional studies, the questions of intervention, blinding, etc. in this tool were considered inappropriate for the analysis, we modified to the following two questions: (1) Was a definition presented before the questionnaire?; and (2) Was the sample randomly obtained? Two researchers (CL and PW) evaluated the studies individually and then compared their results. Each article received an overall score (poor, fair, or good) according to the assessment tool.

### Coding decisions

Depending on the design of the study, some studies reported two groups of victims (TB and CB) [21, 25–46]. Other studies reported victims who only suffered TB (TB only) or CB (CB only), and victims of both types of bullying (Both) [15, 47–64]. For studies with the same source of data, we retained the one with more statistical information to ensure that each effect size was represented only once in the analysis. For longitudinal studies that reported multiple time nodes, we selected the one with more statistical information or larger sample size.

The odds ratios (ORs) and Pearson correlation coefficients were the most reported effect size for mental health problems. In our study, ORs were used as effect sizes. Studies with Pearson correlation or regression coefficients were converted to ORs using an approach similar to that used in van Geel et al. [65]. The experimental groups of ORs were the bullying victims; the control groups were the participants who had not been bullied or not suffered this type of bullying. Some articles categorized bullying into different subtypes of traditional victimization (e.g., physical, relational) [15, 26, 28, 30, 33, 36, 37, 58, 59], in these studies the queried data from the author or the highest prevalence types were used to estimate the effect size. The crude ORs and the adjusted ORs were suitable.

We coded multiple variables regarding sample characteristics and questionnaire design for moderator analysis. The randomization (random vs. non-random) and the institutions included in the sample (single vs. multi-institution) were coded. The source (country) of the sample, age distribution, year of sampling, and grade information were also coded (regions were categorized as Europe and Australia, North America, Asia, and the Middle East, no African and South American study completed our inclusion criterias). The national income (high-income vs. non-high income) based on the World Bank Country and Lending Groups [66] were estimated. Regarding the questionnaire design, some studies just asked about bullying using the choices “Yes” and “No”, while others offered several options (e.g., “Never”, “sometimes”, “often”, “always”); we coded this as an answer setting (Yes/No vs. multiple selections). The time frame for bullying, the frequency of bullying definition (being bullied once was considered a victim versus requiring repeated actions), and the perpetrator in the questionnaire (some studies asked whether the participant also bullied others or not) were coded. All included studies were coded by three authors (PW, CL and MM) independently; the differences were resolved through discussion. The first time the identical rate was around eighty percent.

### Statistical analyses

Given the results of previous studies [10, 67], the random-effects model is a reasonable choice for our study. For the prevalence analysis, a generalized linear mixed model (GLMM) method [68] and Knapp-Hartung adjustment [34]were used to fit the logit transformed effect sizes [69]. For an easy explanation, the effect sizes were then transformed back into proportions when plotting and reporting the results.

Studies with the three groups of victims (TB only, CB only, and Both) and two-group of victims (TB and CB) were compared in a subgroup analyses. The moderator analysis based on a method based on the classification and regression tree (meta-CART) [70] and three-level mixed-effects model [71] were conducted.

Odds ratios are effect sizes for mental health problems, the mean odds ratios were estimated only when more than five studies reported that factor, and the moderator analyses were explored when that factor had no less than 30 effect sizes. Studies with three victim groups (TB only, CB only and Both) and two victim groups (TB, CB) were pooled to estimate the mean effect sizes. The ORs were transformed to log-odds for analysis, then inversed the result to normal ORs for an easy explanation. Studies were performed using the Mantel–Haenszel method [72, 73] when the number of events in each group was provided, then combined with pre-calculated ORs, the inverse-variance method [74] was used to estimate the final results.

Cochran’s Q [75] and $I^{2}$ [76] were reported as the main heterogeneity measures, some analyses also report the prediction interval recommended by [77].The publication bias was analyzed by funnel plot and Egger’s regression test [78]. The R packages “metafor” [79], “meta” [80], “esc” [81], “metacart” [70] and R version 4.1 [82] were used in our meta-analysis.

## Results

### Prevalence rate

**Fig. 2:**
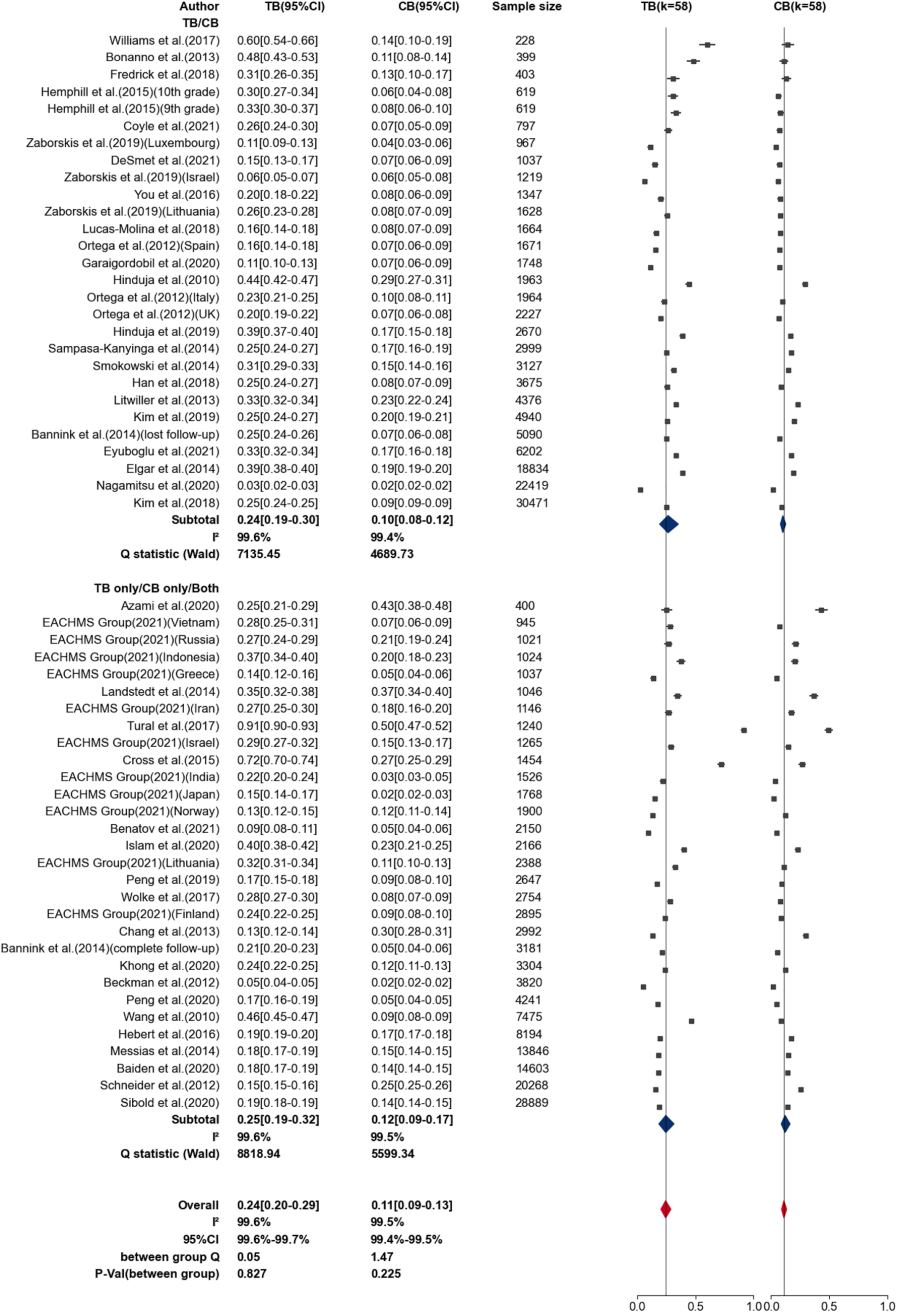
General and subgroup prevalence forest plots of victims of traditional and cyberbullying. *TB* traditional bullying, *CB* cyberbullying. The upper cluster included studies just classified in two groups (TB and CB), the bottom cluster included studies classified in three groups (TB only, CB only and Both). All Q statistics significant at *p* <0.0001

A total of 42 studies with 266,888 participants were used to estimate the prevalences of TB and CB. The mean victimization rate was 24.32% (95% CI 20.32–28.83%) for TB and 11.10% (95% CI 9.12–13.44%) for CB (see Fig. 2). The prediction interval was 5.18–65.42% for TB, and 2.31–39.73% for CB. Subgroup analysis were conducted to compare studies with three groups of victims (TB only, CB only, and Both) and two groups of victims (TB and CB). Cochran’s Q and $I^{2}$ indicated substantial heterogeneity across all subgroups. No significant difference between the the two subgroups was detected, in both TB prevalence ($Q_\text {between} = 0.05$, $p=0.83$)and CB prevalence ($Q_\text {between}= 1.47$, $p=0.23$).

**Fig. 3:**
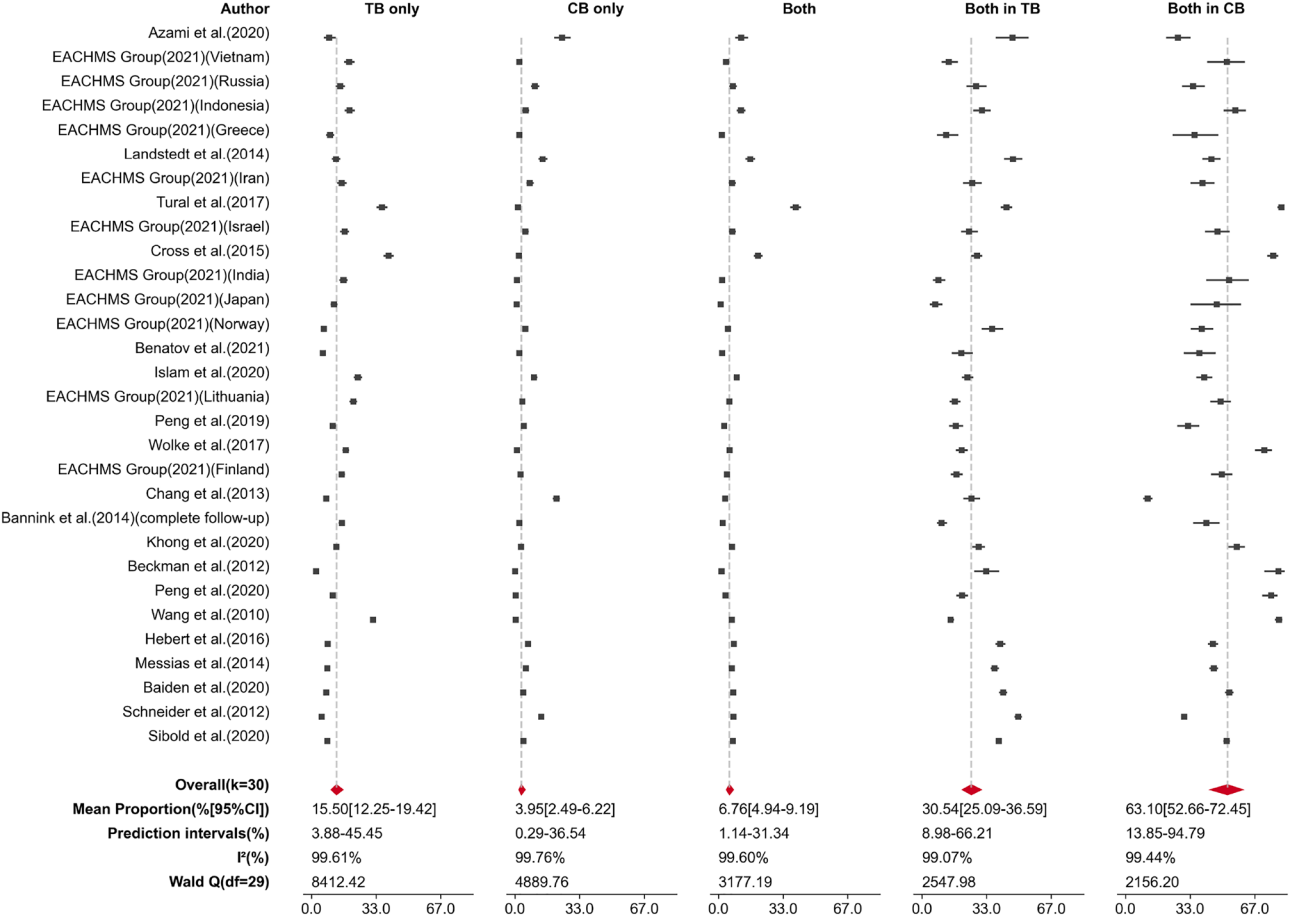
Forest plot of the prevalence of the three groups of victims (column “TB only,” “CB only,” and “Both”) and the mean estimated proportions of the victims of traditional bullying who also experienced cyberbullying (column “Both in TB”), and the victims of cyberbullying who also experienced traditional bullying (column “Both in CB”). All Q statistics significant at *p* <0.0001

As shown in Fig. 3, thirty effect sizes reported three groups (TB only, CB only, and Both) of victims. We estimated the prevalence of each group, with 15.50% (95% CI 12.25–19.42%) for TB only and 3.95% (95% CI 2.49–6.22%) for CB only. Approximately 6.76% (95% CI 4.94–9.19%) experienced both types of bullying. Through these studies with three groups of victims, there were 30.54% (95% CI 25.09–36.59% ) of victims who experienced TB who also cyberbullied, and 63.10% (95% CI 52.69–72.45% ) of victims who experienced CB who had also been bullied in school (TB) (the “Both in TB” and “Both in CB” columns in Fig. 3). The funnel plot and Egger’s regression test did not detect publication bias in the prevalence analysis.

### Moderator analysis

In consideration that the moderator effects may be masked in multilevel interactions, a three-level mixed model and meta-CART were used to look into the feasible multi-level moderator. The results showed that region ($X^2=5.15,p=0.01$) and sampling year ($X^2=2.76,p=0.05$) were significantly better fit compared to two-level models without inter-cluster level. Regional diversity explained 22.8% of $I^2$ and sampling year explained 27.14% of $I^2$(see Table 1). In a subgroup analysis of the prevalences of TB and CB in different regions, the between-group differences were significant in both TB (p=0.05) and CB(<0.001) (Table 1).

**Table 1:** Three-level nested variance component analysis of prevalence of traditional bullying and cyberbullying

	Traditional	Cyber	Traditional only	Cyber only	Both
k	58	58	30	30	30
Q	16098.22 (*df* = 57)	10835.97 (*df* = 57)	8412.42 (*df* = 29)	4889.76 (*df* = 29)	3177.19 (*df* = 29)
$I^2$	99.82%	99.72%	99.62%	99.76%	99.62%
*Region*					
Effect size (96% IC)	0.24 (0.19–0.30)	0.11 (0.08–0.17)	0.16 (0.12–0.20)	0.04 (0.03–0.06)	0.07 (0.05–0.10)
$I^2_{\text {level}_{2}}$ (within-cluster)	94.97%	77.44%	99.62%	99.76%	90.87%
$I^2_{\text {level}_{3}}$ (between-cluster)	4.85%	22.28%	–	–	8.75%
$X^2$ (*p* value)	0.48 (0.24)	**5.15 (0.01)**	–	–	0.35(0.28)
*Sample year*					
Effect size (96% IC)	0.25 (0.20–0.29)	0.12 (0.09–0.15)	0.15 (0.12–0.20)	0.04 (0.02–0.07)	0.07 (0.05–0.10)
$I^2_{\text {level}_{2}}$ (within-cluster)	96.64%	72.57%	84.07%	86.43%	86.06%
$I^2_{\text {level}_{3}}$ (between-cluster)	3.18%	27.14%	15.56%	13.33%	13.56%
$X^2$ (*P* value)	0.04 (0.42)	**2.76 (0.05)**	0.29(0.59)	–	0.29 (0.59)
*Answer setting*					
Effect size (96% IC)	0.23 (0.17–0.31)	0.11 (0.09–0.15)	0.15 (0.10–0.21)	0.05 (0.02–0.10)	0.07 (0.05–0.09)
$I^2_{\text {level}_{2}}$ (within-cluster)	94.76%	97.23%	93.25%	86.96%	99.61%
$I^2_{\text {level}_{3}}$ (between-cluster)	5.07%	2.47%	6.39%	12.82%	–
$X^2$ (*P*-value)	0.18 (0.34)	0.07 (0.40)	0.11 (0.37)	0.40 (0.26)	–

Bold values in the table indicate that the three-level model provides a significantly better fit than the two-level model that constrains level 3 heterogeneity to zero

The lack of results in the table indicate that $I^2$ cannot be explained at this level in the model, or that $X^2$ is equal to 0 and the *p* value is equal to 1

All Q statistics significant at *p* <0.001. All effect size significant at *p* <0.001

For further analysis, the moderator analyses based on three-level models were processed to explore the potential moderator factor (Table 2). The income is a moderator significant after adjusting the effect of regions, in TB ($\beta =-0.86,F=6.86,p=0.01$) and CB($\beta =-0.69,F=5.24,p=0.03$), high-income countries have a lower prevalence of TB and TB than non-high-income countries (Table 2). Asia($\beta =-0.56,F=4.04,p=0.05$) and North America($\beta =0.52,F=5.04,p=0.03$) are significative in CB, Asia had a prevalence 5.10% lower than others regions, and North American had a prevalence 5.90% higher than others regions. Another moderator analysis was applied after adjusting the variation of sampling year (Table 3).

**Table 2:** Univariate moderator analyses about the effects of prevalence by questionnaire design and sample features based on different regions

	Traditional^1^				Cyber^2^			
	Q (*df* = 56)	Beta (SE)	*F* (*df* = 1.56)	*p* value	*Q* (*df* = 56)	Beta (SE)	*F*(*df* = 1.56)	*p* value
Age	14855.71	− 0.17(0.11)	2.50	0.12	9060.65	0.12(0.10)	1.42	0.24
*Questionnaire*								
Answer setting	12756.51	0.63(0.34)	3.53	0.07	10503.25	− 0.14(0.30)	0.21	0.65
Frequency	16056.91	− 0.07(0.24)	0.08	0.78	10370.75	− 0.03(0.22)	0.02	0.90
*Time frame*								
$\le$3 months	15014.28	− 0.06(0.25)	0.06	0.81	10591.49	− 0.04(0.24)	0.03	0.87
6 months	16051.48	− 0.13(0.25)	0.26	0.61	10001.16	− 0.01(0.23)	< 0.01	0.95
12 months	15996.90	0.02(0.27)	<0.01	0.93	9363.67	0.34(0.24)	1.89	0.17
Unlimited	15212.22	0.41(0.41)	1.02	0.32	10770.80	− 0.36(0.28)	1.70	0.20
Bully	14759.50	− 0.01(0.26)	0.01	0.96	10578.63	− 0.19(0.23)	0.72	0.40
Multi-school	15966.45	− 0.81(0.45)	3.22	0.08	10802.36	0.43(0.43)	1.00	0.32
Random	16086.37	0.06(0.25)	0.05	0.82	10449.48	0.21(0.22)	0.92	0.34
Sample year	15432.86	− 0.11(0.12)	0.90	0.35	9594.36	0.01(0.11)	0.02	0.90
Income	15982.14	− 0.86(0.33)	6.86	0.01	10815.82	− 0.69(0.30)	5.24	0.03

^1^Traditional bullying

^2^Cyberbullying

All Q statistics significant at *p*<0.0001

**Table 3:** Univariate moderator analyses about the effects of prevalence by questionnaire design and sample features based on sampling years

	Traditional^1^				Cyber^2^			
	*Q* (*df* = 56)	Beta (SE)	*F* (*df* = 1.56)	*p* value	*Q* (*df *= 56)	Beta (SE)	*F* (*df* = 1.56)	*p* value
Age	14855.71	− 0.15(0.12)	1.70	0.20	9060.65	0.14(0.11)	1.66	0.20
*Questionnaire*								
Answer setting	12756.51	0.44(0.33)	1.73	0.19	10503.25	− 0.26(0.28)	0.88	0.35
Frequency	16056.91	− 0.12(0.25)	0.24	0.62	10370.75	− 0.28(0.23)	1.49	0.23
*Time frame*								
$\le$3 months	15014.28	− 0.04(0.26)	0.02	0.89	10591.49	0.16(0.25)	0.38	0.54
6 months	16051.48	− 0.19(0.25)	0.56	0.46	10001.16	− 0.16(0.24)	0.45	0.50
12 months	15996.90	0.09(0.26)	0.13	0.72	9363.67	0.35(0.24)	2.18	0.15
Unlimited	15212.22	0.40(0.42)	0.91	0.34	10770.80	− 0.50(0.30)	2.78	0.10
Bully	14759.50	− 0.07(0.26)	0.08	0.78	10578.63	− 0.39(0.24)	2.61	0.11
Multi-school	15966.45	− 0.81(0.45)	3.20	0.08	10802.36	0.06(0.42)	0.02	0.89
Random	16086.37	0.08(0.25)	0.12	0.73	10449.48	0.27(0.22)	1.41	0.24
Income	15982.14	− 0.56(0.31)	3.39	0.07	10815.82	− 0.50(0.28)	3.07	0.09
*Region*								
Europe*	16095.84	− 0.18(0.24)	0.57	0.45	10328.51	− 0.38(0.22)	2.91	0.09
Asia	14862.62	− 0.49(0.30)	2.65	0.11	10097.20	− 0.56(0.28)	4.04	0.05
North America	15888.28	0.42(0.25)	2.88	0.10	10219.01	0.52(0.23)	5.04	0.03
Middle East	15887.44	0.26(0.36)	0.50	0.48	10620.44	0.57(0.32)	3.10	0.08

^1^Traditional bullying

^2^Cyberbullying

*The studies of Australia and New Zealand are included in the European data

All Q statistics significant at *p*<0.0001

Overall, income and regions were factors that affected the prevalence of bullying. The high-income countries may have a lower prevalence of both TB and CB than non-high-income countries. The prevalence of TB and CB in Asia was lower than in North America, Europe and the Middle East. In the analysis of the meta-CART method, no tree-like multi-level moderators were found.

### Mental health problems analysis

**Fig. 4:**
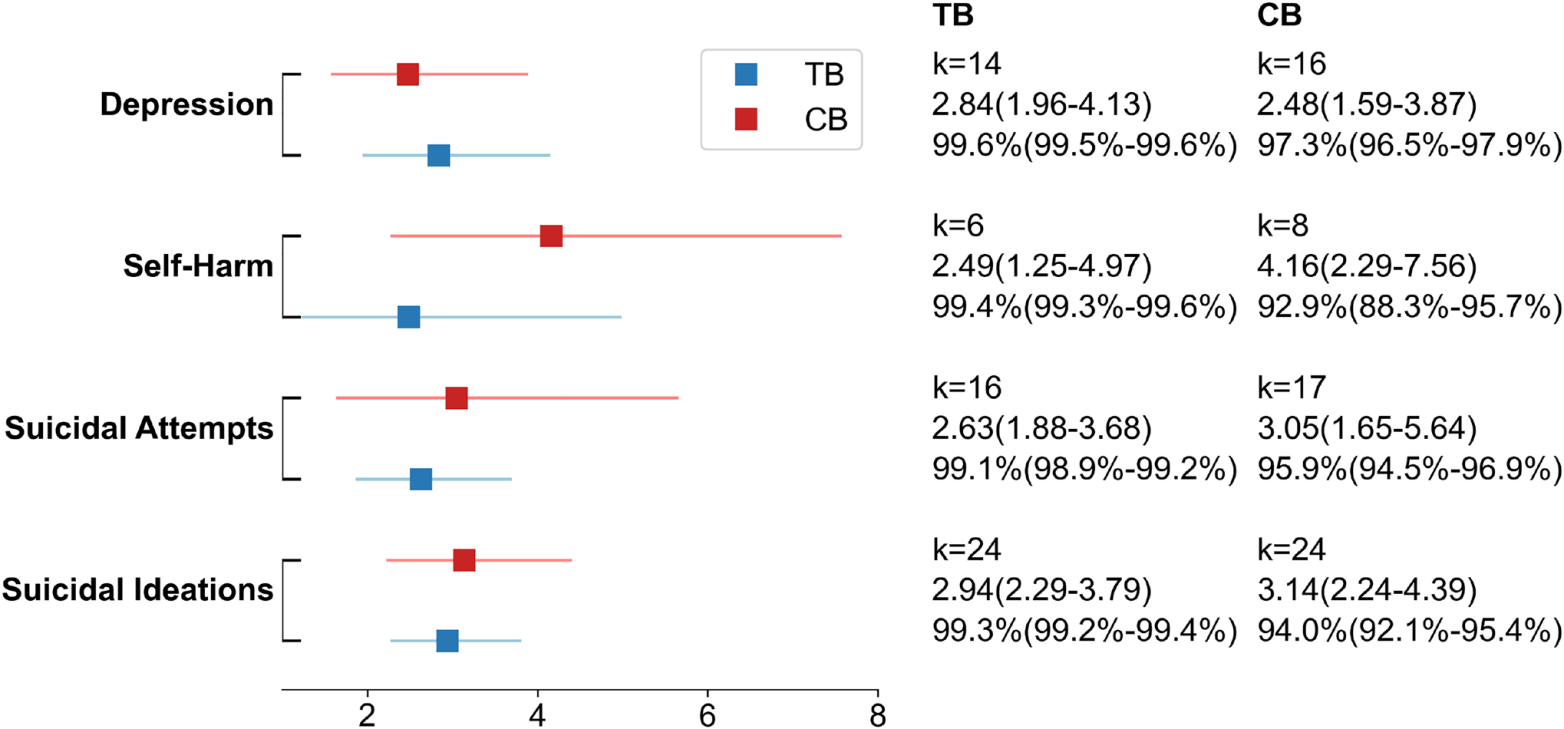
The plot showed the estimated ORs of the main psychological problems in studies reported in a two groups (TB and CB) design. The OR and 95% CI, the number of effect sizes, $I^2$ and its 95% CI were shown on the plot. All results were based on the random-effect model and significant at *p*<0.001

The estimated ORs of the relationship between the psychological problems (depression, suicidal ideation , self-harm and suicide attempts) and either TB or CB are displayed in Fig. 4.

**Fig. 5:**
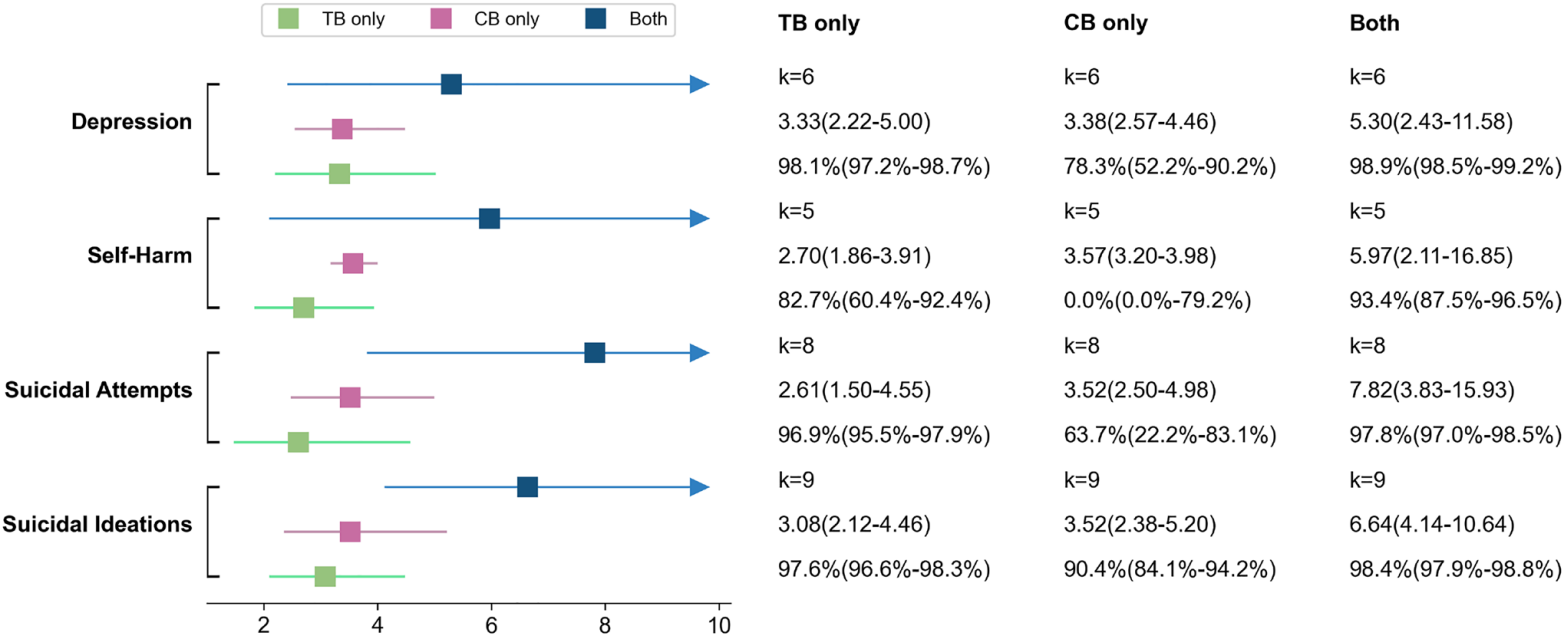
The plot showed the estimated ORs of the main psychological problems in studies reported in a three-group (TB only, CB only and Both) design. The OR and 95% CI, the number of effect sizes, $I^2$ and its 95% CI were shown on the plot. All results were based on the random-effect model and significant at *p* <0.01

Even though fewer studies reported three groups of victimization (TB only, CB only, and Both), the core mental health problems still had more than five studies. Across the results, the ORs in the three groups were even higher than in the two groups (TB and CB), this is due to the proportion of crude ORs increased in the three groups analysis. The crude OR is generally slightly higher than the adjusted OR. The result was hard to compare directly to the two groups. However, it still was easy to compare within the three groups of bullying victims. For all mental health problems (suicidal ideation, suicidal attempts, self-harm, and depression), the ORs were much higher in victims with experience of both types of bullying compared to the other two groups of victims, even though the overleaped zones were still obviously. The ORs of CB only were slightly higher than TB only but with the zones overleaped (see Figs. 4 and 5).

The ORs of anxiety were also collected, but few studies reported three groups (TB only, CB only and Both), only four studies reported the two groups (TB and CB), which showed the mean OR of anxiety was 2.37 (95% CI0.82–6.83, *p*=0.08) in TB, and 3.04 (95% CI2.82–3.27, $p<0.0001$) in CB. Some studies also focus on psychiatric symptoms measured by the Strengths and Difficulties Questionnaire (SDQ). However, most of them duplicated the samples with the study published by the Eurasian Child Mental Health Study (EACMHS) Group [50], with no change in conclusions.

Our findings revealed that bullying victims have a higher risk for psychological problems than non-bullied people, that CB victims have a slightly higher risk than TB victims, and that victims who have experienced both TB and CB have a higher risk than victims who have only experienced one type of bullying. However, the overlap of confidence intervals between different types of bullying makes the results ambiguous. Nevertheless, we did not detect publication bias, except in the analysis about the OR of depression in TB (*t*=2.54,*p*=0.03).

## Discussion

This meta-analysis aimed to explore the association between either TB and/or CB, and related mental health risks. Our meta-analysis showed notable findings, enumerated as follows. (1) The prevalence of TB victims was about twice that of CB victims. About one-third of youth who suffered TB also suffered CB. Conversely, two-thirds of youth who suffered CB also experienced TB. (2) Region is a robust moderator for prevalences, especially for CB. (3) High-income countries appear to have lower prevalences of TB and CB than non-high-income countries. (4) Suicidal ideations, suicide attempts, and self-harm are at higher risk with CB than with TB. (5) Victims who experienced both TB and CB had a substantial increase in both suicide-related and depression risks.

In the current study, the mean victimization rate for TB was 24.32%, and 11.10% for CB. These figures are slightly lower than those reported in a meta-study published in 2014 reporting a victimization rate for TB and CB of 36%, and 15%, respectively [5]. These differences may be affected by the proportion of studies from Asia, which had lower bullying rates than other regions. The bullying rate in North America (30% for TB and 15% for CB in our study) are pretty close to the figures reported in the previous study. The previous studies [10, 11] had pointed out that the prevalence of cyberbullying in Europe and America was different and considered that it was affected by culture and policy factors. We added the studies from Asia and the Middle East, and this difference is especially pronounced in CB. The culture and politics may have a more pronounced impact on online behavior and performances.Another relevant finding was that income may influence the prevalence of bullying, as the prevalence of TB and CB victimization rate tends to be lower in high-income countries. This result is consistent with the finding of a study about socioeconomic status and bullying in 2014 by Tippett and Wolke [83]. The answer setting and the number of institutions included in the samples did not reach significant results in the moderator analysis, they may have a weak moderator function but were concealed by the insufficient of effect sizes. The study [5] reported that the questionnaire included the questions of perpetrators and the random setting of the studies were moderator variables. However, these two factors were not significant in our study. The random error in the random-effect models could make the weak moderators fewer effects.

One of the purposes of our study was to explore whether bullying prevalence has changed over the last decade when youths worldwide have widely used mobile devices extensively. Although the sampling year can explain a certain amount of inter-cluster variation in the three-level model analysis, it is not meaningful as a moderating variable, in either TB or CB. Three studies [61, 62, 64] that used the Youth Risk Behavior Surveillance System (YRBSS) showed no changes in either TB or CB victim rates from 2011 to 2017. Despite the increased awareness of bullying in American society over the past decade, there is still no measure that can be taken to reduce bullying rates, and the popularity of mobile phones seems not to lead to an increase in CB over the years. Another finding in our study showed that two-thirds of CB victims also experienced TB, the ratio is slightly higher than in another earlier study [11], which could be related to the fact that young people have spent more time online in recent years, and that traditional forms of bullying are expanding online.

Because we required the included studies to report TB and CB prevalence while focusing on at least one mental health risk, this ensured that most of the inter-bullying-group ORs were estimated based on the same samples. In the analyses, we confirmed the increase in risks of suicidal ideation, suicide attempts, self-harm and depression for TB and CB compare to the non-victims of bullying. Some studies also showed similar conclusions [22, 65, 84]. When we compared the studies with two victim groups(TB and CB) and the three groups (TB only, CB only and Both), except for the ORs of depression, the suicide/self-injury related risks were higher in CB than in TB. A study from Germany also showed that bullying based on social relationships and networks has a greater negative psychological impact on young people than physical bullying [85]. Moreover, our studies showed that the youth who had been bullied by TB and CB had much higher risks than those who just had experienced one type of bullying. Furthermore, most youths with CB are also victims of TB. Thus, CB may be a marker of poor real-world socialization [14, 86].

In cyberbullying, the perpetrator could be anonymous, the public tends to have lower guilt and ethic on the Internet, also the function of diffusion and storage make the victim hard to predict and control the influence and duration of the bullying [10]. In traditional bullying, the participants are usually limited and the perpetrators are clearly identified, and much easy to anticipate and avoid the bullying. This could be the point why the CB victims showed higher risks of those mental health problems. Even though TB still predominates over CB, based on the current trend, the overlap between TB and CB may become more common. Online and offline bullying concurrence could cause severe consequences. For education and social regulators, it may be necessary to explore and formulate policies specifically to protect victims of CB, especially in areas where TB cultures are more prevalent and in developing countries.

Our study also has some limitations. First, most of the articles included in our study were cross-sectional studies, so we cannot make causal statements about the relationships between TB/CB and psychological variables. The inclusion of more longitudinal studies to explore possible causal relationships is recommended in the future. Second, all articles collected data in a self-report form and may be subject to recall bias. On the other hand, each study used different scales. For example, some studies used single-item measures, while others used multiple checklists to obtain data; some articles defined bullying before completing the scale, while others did not; also, some studies used questionnaires which did not mention the word ”bullying” to avoid re-traumatizing victims of bullying. The above problems may lead to some unavoidable bias in our results. Third, the use of secondary data limited our ability to examine other outcome-related factors, such as household income, individual mental health, academic satisfaction, and family and social support. Fourth, previous literature has shown that the impact depends on the type of bullying. For example, some types of CB (e.g., insults,threats) is considered less harmful than TB, while those that use images or videos are considered more harmful [4]. Fifth, the data obtained were insufficient for some analyses, we could not analyze the effect of potential moderating variables. Some studies [46, 58, 63, 87, 88] showed that age, gender, subtype, social support, race, relationship with parents, and sexual orientation have moderating effects. Finally, our research has focused only on the victims of bullying,and this is a dyadic problem including both bullies and victims. Thus some victims are also perpetrators [5, 10]. Therefore, we need to explore the above issues in future research to understand bullying better and help prevent it from occurring.

## Conclusion

This meta-analysis establishes that TB and CB victimization among youth are a matter of public health concern. The measures implemented in the last decade may not have reduced the occurrence of TB and CB. Victimization appears to be a marker of greater psychopathological severity, particularly suicide-related issues. In the mobile and streaming era in which we live, more studies that explore the impact of peer bullying are indispensable in the development of public policies devoted to mitigating the impact of both TB and, particularly, CB.

## Electronic supplementary material

Below is the link to the electronic supplementary material.

Supplementary file1 (PDF 140 kb)

## Data Availability

All data used in the meta-analysis can be found in the included studies. Supplementary data related to this article can be found at https://osf.io/hk8dv/?view_only=a028bcc17e8945cb8e04148e38f05826.

## Code availability

Analysis codes are available on request from the corresponding author.
