# Supplementary material for: Traditional bullying and cyberbullying in the digital age and its associated mental health problems in children and adolescents: a meta-analysis
Source: Eur Child Adolesc Psychiatry. 2022 Dec 31;33(9):2895–909. doi: 10.1007/s00787-022-02128-x (PMC11424704; doi:10.1007/s00787-022-02128-x)
Supplement: Supplementary file 1 — Supplementary file1 (PDF 140 kb) [file 787_2022_2128_MOESM1_ESM.pdf]

Author/Date	Country	Study Design	Site of data collection	Sample size	Gender (% Female)	Mean Age (SD) OR Range (yrs)	Traditional bulllying measure	Cyberbulllying measure	SI measure	SA measure	Self-harm measure	Depression measure	Anxiety measure	Self-Esteem measure	Other Mental health measure	Quality rating
Benatov et al. 2021	Israel	LS	9th to 12th grade students	2150	21,3%	15,42 (0,97)	Single item	Single item	PSS	PSS	\	BSI	\	\	\	Fair
DeSmet et al. 2021	Belgium	CS	7th to 12th grade students	1037	50,0%	15,2 (1,9)	Single item	Single item	DASS-21	DASS-21		DASS-21	DASS-21		DASS-21	Good
Eyuboglu et al. 2021	Turkey	CS	6th to 12th grade students	6202	46,0%	14,4 (1,9)	YRBSS	YRBSS	\	\	YRBSS	HADS	HADS	RSES	SDQ	Good
Coyle et al.2021	USA	CS	7th to 8th grade students	799	48,2%	Not stated	MCVS	MCVS	\	\	\	BASC-2	BASC-2	\	BASC-2	Good
EACHMS Group 2021	Japan, Greece, Norway, India, Finland, Vietnam, Israel, Iran, Lithuania, Russia, Indonesia	CS	Not stated	21688	47,3-54,7%	Range 13-15	Single item	Single item	\	\	\	\	\	\	SDQ	Good
Peng et al. 2020	China	CS	7th to 12th grade students	4241	44,2%	14,36 (1,8)	JCVQ	JCVQ	JCVQ	JCVQ	JCVQ	\	\	\	K-10	Good
Nagamitsu et al.2020	Japan	CS	7th to 9th grade students	22419	48,0%	Range 13-18	Single item	Single item	Single item	Single item	\	\	\	\	Single item	Poor
Islam et al.2020	Australia	CS	7th to 12th grade students	2166	47,8%	14,83 (1,7)	YRBSS	YRBSS	YRBSS	YRBSS	YRBSS	DISC IV	DISC IV	\	DISC IV	Good
Sibold et al.2020	USA	CS	9th to 12th grade students	29207	Not stated	Rang 14-18	YRBSS	YRBSS	YRBSS	YRBSS	\	\	\	\	YRBSS	Poor
Garaigordobil et al. 2020	Spain	CS	7th to 12th grade students	1748	52,6%	Range 13-17	SPH	\	\	\	\	BDI-II	SAS-A	\	SCL-90R	Fair
Baiden et al. 2020	USA	CS	9th to 12th grade students	14603	52,0%	Range 14-18	YRBSS	YRBSS	YRBSS	YRBSS	\	YRBSS	\	\	YRBSS	Good
Azami et.al 2020	Iran	CS	Not stated	425	53,2%	16,61 (0,95)	OB/VQ	E-VS	\	Single item	Single item	\	\	\	Single item	fair
Peng et al.2019	China	CS	7th to 9th grade students	2647	51,2%	13,6 (1,1)	Single-item	Single-item	Single-item	Single-item	Single-item	\	\	\	SDQ	Good
Khong et al. 2020	Singapore	CS	7th to 11th grade students	3329	49,8%	14,4 (1,5)	Single-item	Single-item	\	\	\	\	\	\	SDQ	Good
Kim et al. 2019	Canada	CS	7th to 12th grade students	4940	56,7%	15,19 (1,8)	Single-item	Single-item	Single-item	\	\	Single-item	Single-item	\	Single-item	Good
Zaborskis et al. 2019	Israel, Lithuania, Luxembourg	CS	7th to 12th grade students	3814	47,7-54,7%	Rang 15-16	Single-item	Single-item	YRBSS	\	YRBSS	\	\	\	\	Good
Hinduja et al.2019	USA	CS	7th to 12th grade students	2670	49,9%	14,5	Multi-item	Multi-item	NASHS	NASHS	\	\	\	\	\	Good
Lucas et al.2018	Spain	CS	Not stated	1664	53,0%	16,12 (1,36)	BCB	BCB	PSS	PSS	\	\	\	\	\	Fair
Han et al. 2018	China	CS	4th to 12th grade students	3675	51,8%	13,6 (2,5)	SSCS	SSCS	YRBSS	YRBSS	\	\	\	\	\	Fair
Fredrick et al. 2018	USA	CS	9th grade students	403	50,0%	14,5 (0,5)	OB/VQ	CBVS	SIQ-JR	\	\	CDI-2	\	\	\	Good
Kim et al 2018	Canada	CS	6th to 12th grade students	31148	51,9%	13,52 (2,04)	OMESS	OMESS	\	\	\	\	\	\	EPS	Good
William et al. 2017	USA	CS	9th to 12th grade students	233	55,0%	14,5 (0,5)	BS	Single-item	Single-item	Single-item	\	CDSD-10	\	\	\	Fair
Wolke et al. 2017	UK	CS	Not stated	2745	56,9%	13,5 (1,35)	BFI	BFI	\	\	\	\	\	SDQ	SDQ	Good
Tural et al. 2017	Turkey	CS	Not stated	1276	49,3%	Not stated	PBRS-AF	CBS	\	\	\	BSI	BSI	BSI	\	Fair
Hébert et al.2016	Canada	LS	10th to 12th grade students	8194	57,8%	Range 14-18	Single-item	Single-item	Single-item	\	\	\	\	SDQ	K-10	Good
You et al. 2016	South Korea	CS	7th to 9th grade students	1347	48,5%	Not stated	CBVS	CBVS	\	\	\	CES-D	\	SES	\	Poor
Cross et al. 2015	Australia	LS	8th grade students	1504	53,0%	Range 13-15	OB/VQ	OB/VO	\	\	\	Single-item	\	\	SDQ	Fair
Hemphil et al. 2015	Australia	LS	9th to 11th grade students	927	54,0%	15,1 (0,4)	Multi-item	Multi-item	\	\	Single-item	SMFQ	\	\	\	Good
Elgar et al. 2014	USA	CS	7th to 12th grade students	18834	50,7%	15 (1,7)	UIAS	UIAS	UIAS	UIAS	UIAS	UIAS	UIAS	\	\	Good
Messias et al. 2014	USA	CS	9th to 12th grade students	15425	Not stated	Range 12-18	YRBSS	YRBSS	YRBSS	YRBSS	\	YRBSS	\	\	\	Fair
Bannik et al. 2014	Netherlands	LS	7th grade students	3181	49,0%	12,5 (0,62)	Single-item	Single-item	Single-item	\	\	\	\	\	SDQ	Poor
Landstedt et al. 2014	Sweden	CS	7th to 9th grade students	1214	52,7%	Range 13-16	Single-item	Single-item	\	\	\	CES-D	\	\	\	Fair
Smokowski et al. 2014	USA	LS	6th to 8th grade students	3127	52,2%	12,7	SSP+	SSP+	\	\	\	YSR	YSR	YSR	YSR	Poor
Sampasa et al. 2014	Canada	CS	7th to 12th grade students	3509	54,9%	14,5 (1,8)	Single-item	Single-item	YRBSS	YRBSS	\	YRBSS	\	\	\	Good
Bonanno et al. 2013	Canada	CS	8th to 10th grade students	399	57,1%	14,2 (0,91)	OB/VQ	OB/VO	SIQ-JR	SIQ-JR	SIQ-JR	CES-D	\	\	\	Good
Litwiller et al. 2013	USA	CS	Not stated	4693	47,0%	16,11 (1,2)	YRBSS	YRBSS	YRBSS	YRBSS	YRBSS	\	\	\	YRBSS	Good
Chang et al. 2013	Taiwan	CS	10th grade students	2992	48,0%	Not stated	YRBSS	YRBSS	\	\	\	CES-D	\	\	RSS	Fair
Ortega et al.2012	Spain, UK, Italy	CS	Not stated	5862	48,8%	14,2 (1,77)	DAPHNE	DAPHNE	\	\	\	\	\	\	DAPHNE	Good
Beckman et al. 2012	Sweden	CS	7th to 9th grade students	3820	51,1%	Range 13-16	OB/VQ	OB/VO	\	\	\	\	\	\	PSP	Good
Schneider et al.2012	USA	CS	9th to 12th grade students	20406	50,4%	Not stated	YRBSS	YRBSS	YRBSS	YRBSS	YRBSS	YRBSS	\	\	\	Good
Wang et al. 2010	USA	CS	6th to 10th grade students	7475	51,5%	14,2 (1,42)	OB/VQ	OB/VO	\	\	Single-item	Multi-item	\	\	\	Fair
Hinduja et al.2010	USA	CS	6th to 8th grade students	1963	50,1%	12,8 (1,12)	Multi-item	Multi-item	NASHS	NASHS	\	\	\	\	\	Good

% = percent; yrs = years; SI = suicide ideation; SA = suicide attempt; LS = Longitudinal study; CS = Cross-sectional study

YRBSS = Youth Risk Behavior Surveillance System; JCVQ = The Juvenile Campus Violence Questionnaire; DISC-IV = Diagnostic interview schedule for children -version IV; PSS = Paykel Suicide Scale; BSI = Brief Symptoms Inventory; SPH: Screening of Peer Harassment; BDI-II = Beck Depression Inventory-II; SAS-A =Socail Anxiety Scale for adolescent; SCL-90-R = 90-Symptom Checklist-Revised; DASS-21 = Depression, Anxiety, Stress scale; SDQ = Strength and Difficulties Questionnaire; RSES = Rosenberg Self-Esteem Scale; MCVS = The Multi-Construct Victim Survey; BASC-2 = The Behavior Assessment System for Children, Second Edition; K-10 = 10-item Kessler Psychological Distress Scale; E-VS = E- victimisation scale; OB/VQ = Revised Olweus Bully/Victim Quistionnarie; NASHS = National Adolescent Student Health Survey; BCB = Brief cyberbullying questionnaire; SSCS = School Survey on Crime and Safety; CBVS = Cyberbullying and Victimization Survey; CDI-2 = Children's Depression Inventory 2nd Edition Short Version; SIQ-JR = Suicidal Ideation Questionnaire-Junior; OMESS = Ontario Ministry of Education's Safe Schools Survey; EPS = Emotional Problems Scale; BS = Bully Survey; CESD-10 = Centers for Epidemiological Studies Depression Survey-10; BFI = Bullying and Friendship Interview schedule; CES-D = Centre for Epidemiologic Studies 20-item Depression Scale (CES-D), SMFQ = Short Mood and Feelings Questionnaire; GBS = Gatehouse Bullying Scale; SMFQ = Short Mood and Feelings Questionnaire; RSS = Rosenberg self-esteem scale; UIAS = The Bullying and Victimization subscales of the University of Illinois Aggression Scales; SSP+ = School Success Profile; YSR= Youth Self-Report(Achenbach and Ruffle's 2000); DAPHNE = DAPHNE Questionnaire [Genta et al., 2012]; PSP = PsychoSomatic Problems scale; CBVS = California Bullying Victimization Scale; SES = Self-Esteem Scale, PBRS-AF = Peer Bullying Rating Scale Adolescent Form, CBS = cyber bullying scale; BSI = Brief Symptom Inventory
